# Comparison of Fecal DNA Extraction Kits for the Giant Panda (*Ailuropoda melanoleuca*) by Short Tandem Repeat Genotype Analysis

**DOI:** 10.1002/ece3.71242

**Published:** 2025-04-08

**Authors:** Jie Gao, Chunhai Li, Yitao Wu, Xinyong Zhu, Siqin Liu, Yang Zhang, Huizhong Pang, Jiaheng Li, Jiawen Liu, Wangsheng Zhao, Ye Wang, Jie Kou

**Affiliations:** ^1^ Sichuan Key Laboratory of Conservation Biology on Endangered Wildlife Chengdu Research Base of Giant Panda Breeding Chengdu Sichuan China; ^2^ College of Life Sciences and Engineering Southwest University of Science and Technology Mianyang Sichuan China

**Keywords:** DNA extraction, fecal samples, giant panda, host DNA, short tandem repeats

## Abstract

Genetic analysis of short tandem repeat (STR) loci using noninvasive fecal samples is currently the most widely used method in genetic surveys of giant pandas ( *Ailuropoda melanoleuca* ). However, low‐quality fecal DNA obtained from fecal samples may affect the accuracy of short tandem repeat (STR) genotyping results and pose a challenge to accurately identify individuals. The aim of this study was thus to compare the efficiency of DNA extraction kits in obtaining high‐quality fecal host DNA from giant panda fecal samples. In this study, six commercial kits widely used in fecal DNA extraction, the QIAamp Fast DNA Stool Mini Kit (Q kit), Beaver Beads Stool DNA Kit (H kit), Mag‐MK Soil & Stool Genome DNA Extraction kit (S kit), Magnetic Soil And Stool DNA Kit (T kit), E.Z.N.A Mag‐Bind Stool DNA Kit (O kit) and Mag Beads Fast DNA Kit for Feces (M kit) were compared. Fecal DNA concentration and purity were measured, and STR genotyping was performed using blood and fecal DNA from captive giant pandas to compare the genotype matches at 11 STR loci. Our results show that the most efficient extraction kits were the Q and T kits, and the Q kit had a greater ability to remove PCR inhibitors than other kits. Careful selection of DNA extraction kits is required to achieve optimal genotyping accuracy across different STR genotyping systems. For STR genotyping systems with smaller PCR product sizes (< 200 bp, such as GPL‐29, GP‐08, GP‐01, Panda‐40 and Panda‐05), all six kits demonstrated high genotype matching rates (GMR > 80%). In contrast, for STR genotyping systems with longer PCR product sizes (> 200 bp), the choice of DNA extraction kit significantly influenced GMR, with the H kit and O kit performing well for gpy‐5 but the Q kit and O kit being less suitable for GPL‐08.

## Graphical abstract

The aim of this study was to compare the efficiency of DNA extraction kits in obtaining high‐quality fecal host DNA from giant panda fecal samples. Our results show that the most efficient extraction kits were the QIAamp Fast DNA Stool Mini Kit and Magnetic Soil And Stool DNA Kit. The present study also emphasizes the need to select the most suitable kit according to the STR genotyping systems used to achieve the highest possible genotyping accuracy.

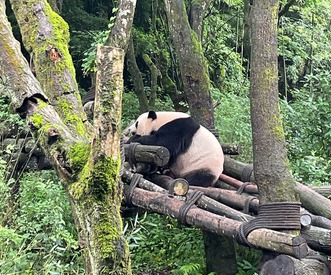

## 1 Introduction

**FIGURE 1:**
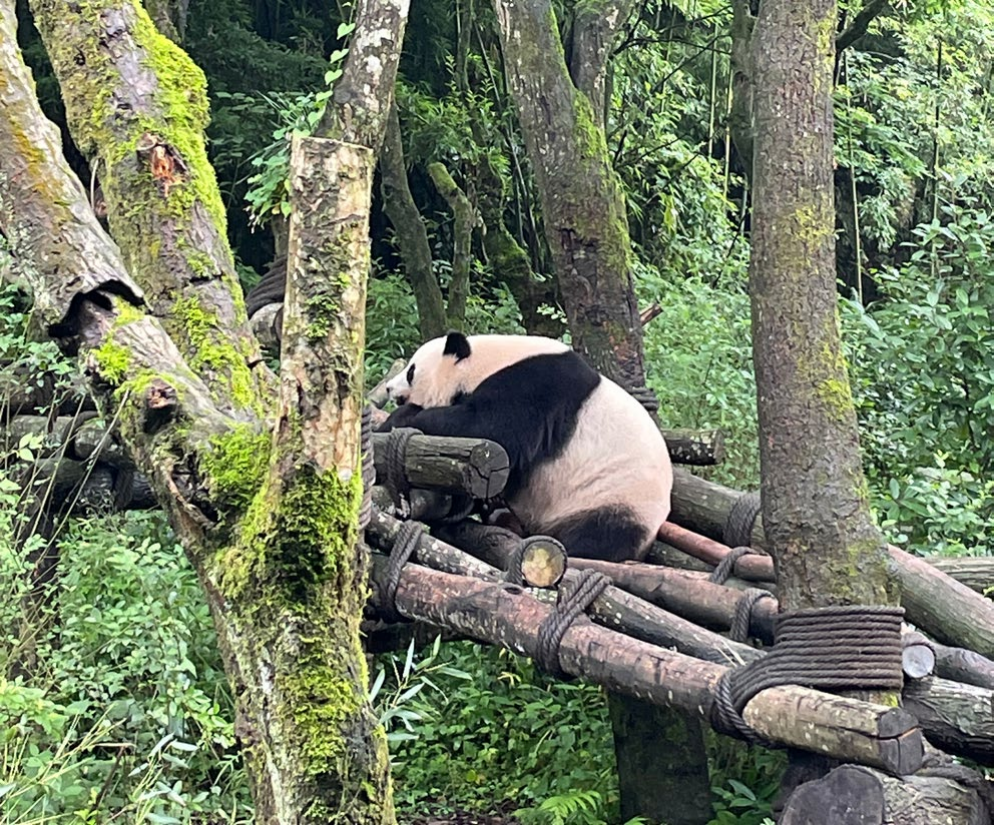
A captive giant panda ( *Ailuropoda melanoleuca* ) at Chengdu Research Base of Giant Panda Breeding.

The giant panda ( *Ailuropoda melanoleuca* ) (Figure 1) is an endemic and endangered species in China and still faces a great risk of extinction. Conservation efforts for the giant panda heavily rely on understanding population dynamics and genetic diversity, which are often assessed through noninvasive samples and a relatively small number of short tandem repeats (STRs or microsatellites) (Zhang et al. 2007; He et al. 2008; Hu et al. 2010; Yang et al. 2011; Wiedower et al. 2012; State Forestry Administration 2021; Ma et al. 2018; Qiao et al. 2019; Dai et al. 2020; Huang et al. 2022; Zhou et al. 2022; Li et al. 2023). Although fecal samples can be collected noninvasively, their DNA quality is usually low and therefore less likely to identify all individuals (Taberlet et al. 1998). According to the results from the fourth national survey of giant pandas, only 473 of the 1308 fresh noninvasive samples were successfully genotyped (samples were considered to have been run successfully if there was amplification at 8 of the 12 STR loci) (State Forestry Administration 2021). In a recent study, a total of 406 out of 971 giant panda fecal samples (no more than 2 weeks) from Liangshan Mountains successfully completed PCR amplification and genotyping for 7 STR loci (Li et al. 2023). In practice, matching of samples to individuals can be problematic due to the complexity and heterogeneity of the fecal composition. Endogenous nucleases from feces and environmental factors such as light, temperature, and humidity can accelerate the degradation of DNA (Fernando et al. 2003; Lonsinger et al. 2015). Moreover, various PCR inhibitors in feces, such as polysaccharides and secondary metabolites of plants, may be co‐extracted with DNA (Deuter et al. 1995; Kohn and Wayne 1997; Monteiro et al. 1997; Wilson 1997). Therefore, the proportion of fecal host DNA is typically low (< 5%), due to the high abundance of exogenous DNA from dietary sources, the gut microbiome, and environmental contaminants (Whitney et al. 2004; Perry et al. 2010; Snyder‐Mackler et al. 2016). These characteristics can dramatically reduce the sensitivity and amplification efficiency of PCR when targeting the host DNA, which may lead to PCR amplification failures or STR genotyping errors (Pierre et al. 1996; Taberlet et al. 1998). When evaluating the quality of fecal DNA using STR genotyping, it is necessary to consider both the host DNA content and the total DNA purity. As a consequence, a reliable genotype at an STR locus using a fecal DNA sample can be quite challenging. For endangered wildlife such as the giant panda, collecting blood or tissue samples can be very difficult. Improving the efficiency in the use of noninvasive samples is almost the only option for genetic surveys of wild giant pandas. To overcome these limitations, researchers suggested that improving the efficiency of DNA extraction, carrying out pilot studies, using a consensus profiling approach for each STR locus, and the use of trinucleotide or tetranucleotide STRs are necessary (Navidi et al. 1992; Pierre et al. 1996; Taberlet et al. 1998; Maudet et al. 2004; Renan et al. 2012). Among these suggestions, DNA extraction is a crucial initial step.

Previous studies have demonstrated that the choice of fecal DNA extraction methods significantly impacts the reliability and accuracy of research outcomes, and it is essential to optimize these methods based on species‐specific characteristics. For example, Hart et al. (Hart et al. 2015) compared five common fecal DNA extraction methods across five species (zebrafish, mice, cats, dogs, and horses) and found that DNA extraction methods significantly affect DNA concentration, purity, NGS (Next‐Generation Sequencing) amplification success rates, and microbial community composition in a species‐dependent manner. Similarly, Videnska et al. (Videnska et al. 2019) reported that different DNA extraction kits can significantly alter the observed composition of fecal bacterial communities following 16S rRNA gene sequencing using the MiSeq Illumina platform, particularly for Gram‐positive bacteria. Furthermore, Nourrisson et al. (2020) showed that different DNA extraction methods and qPCR protocols lead to significant variations in the detection rates of parasites (e.g., *Blastocystis* sp.) in fecal samples. These findings underscore the importance of matching fecal DNA extraction methods with the specific characteristics of the target species' feces. For any given species, it is essential to conduct a targeted selection of fecal DNA extraction kits when considering both DNA quality and the requirements of downstream genetic analyses.

The unique physiological characteristics and dietary habits of giant pandas result in significant differences in their fecal composition compared to other animals. Therefore, selecting appropriate commercial kits for the extraction of high‐quality DNA from panda feces is particularly crucial. Although giant pandas primarily feed on bamboo, their digestive system retains typical carnivore traits, such as the lack of specialized fermentation organs like the cecum and rumen, as well as the absence of cellulase enzymes. This combination of carnivorous digestive structures and herbivorous dietary habits leads to feces primarily composed of undigested bamboo fibers, along with a variety of complex PCR inhibitors (e.g., polysaccharides, polyphenolic compounds) (Yan et al. 2023). These components, which affect fecal DNA extraction, challenge the ability of commercial kits to effectively remove contaminants while obtaining high‐quality target DNA (Friar 2005). Currently, only two commercial kits are widely used in giant panda genetic studies based on STR genotyping. Among them, the QIAamp fast DNA stool mini kit is the most commonly used (Huang et al. 2015, 2022; Yang et al. 2018; Qiao et al. 2019; Dai et al. 2020; Connor et al. 2022; Zhou et al. 2022; Xu et al. 2024), while a magnetic bead‐based kit (Biobase Upure DNA stool kit) only reported in one application case for automated DNA extraction system (Li et al. 2023). However, systematic evaluations of commercially available kits for giant panda fecal DNA extraction are still lacking. Given that many commercial kits claim to efficiently extract fecal DNA, there may be significant differences in their performance in recovering short DNA fragments or removing PCR inhibitors, potentially leading to the loss of host DNA or excessive residual PCR inhibitors (He et al. 2019; Srirungruang et al. 2022). Therefore, conducting comparative studies on different commercial kits is of great significance for optimizing STR genotyping efficiency and improving the quality of giant panda genetic research.

In this study, fresh fecal samples were collected from five giant panda individuals, and thirty DNA samples were extracted by six commercial DNA extraction kits (QIAamp Fast DNA Stool Mini Kit, Beaver Beads Stool DNA Kit, Mag‐MK Soil & Stool Genome DNA Extraction kit, Magnetic Soil And Stool DNA Kit, E.Z.N.A Mag‐Bind Stool DNA Kit and Mag Beads Fast DNA Kit for Feces). The present study compared the DNA extraction performance of these kits with a focus on DNA concentration, DNA purity, genotype matching rate (GMR), amplification success rate (ASR) and the allele peak intensities. In addition, we evaluated the amplification performance of 11 STR genotyping systems by analyzing the total genotype matching rate (TGMR). This comparative study is expected to improve the efficiency of STR genotyping analysis of giant panda fecal samples.

## 2 Materials and Methods

### 2.1 Sample Collection and Preparation

Blood and fecal samples were collected from five healthy captive giant panda individuals housed at the Chengdu Research Base of Giant Panda Breeding. Blood samples were stored in a −80°C freezer. Fresh fecal samples (less than 12 h of exposure) were carefully collected using disposable gloves, preserved in sterile bags, and transported immediately to the laboratory. Sampling and other experiments were approved by the Chengdu Research Base of Giant Panda Breeding Institutional Animal Care and Use Committee (approval numbers 2020015 and 2021006).

Fecal samples were collected during the season when giant pandas primarily consumed bamboo culm and leaves. For each individual, 90 g of fecal sample was mixed in a total of 400 mL phosphate buffered solution (PBS, pH 7.4) and cultured at 2°C–8°C in a rotary shaker (KS4000i, IKA, German) at 120 rpm for 16 h to become homogeneous. After centrifugation (400 *g*, 5 min) to remove large plant residues, the fecal supernatant was divided into six portions (60 mL each) and transferred into 100 mL centrifuge tubes, which were then centrifuged (800 *g*, 10 min). The fecal pellet was collected and weighed for later use.

### 2.2 DNA Extraction

DNA of blood samples was extracted using QIAamp DNA Blood Mini Kit according to the manufacturer's instruction. Thirty fecal DNA samples were isolated from fecal pellets by using six commercial fecal DNA extraction kits (summarized in Table 1), including five commercial magnetic bead‐based DNA extraction kits and one silica membrane‐based DNA extraction kit. These kits were selected because they are widely cited in studies of host DNA or nonhost DNA in giant panda fecal samples, or recommended by the manufacturer to be useful for PCR amplification. For all six extraction kits, fecal DNA extraction was performed according to the manufacturer's instructions, with a standardized elution volume of 600 μL to ensure cross‐kit consistency. For QIAamp Fast DNA Stool Mini Kit (Q kit) and E.Z.N.A Mag‐Bind Stool DNA Kit (O kit), we followed the protocols for host DNA analysis. For kits (S kit, T kit, and M kit) requiring RNase A, we added RNase A (Takara Japan, 10 mg/mL). For Magnetic Soil And Stool DNA Kit (T kit) and Mag Beads Fast DNA Kit for Feces (M kit), we vortexed at maximum speed for sample homogenization according to the manufacturer's instructions. All DNA samples were stored at −20°C after observation and recording of DNA elution color until use.

**TABLE 1:** The six commercial DNA extraction kits used in this study.

Full name of the kit	QIAamp Fast DNA Stool Mini Kit	Beaver Beads Stool DNA Kit	Mag‐MK Soil & Stool Genome DNA Extraction kit	Magnetic Soil And Stool DNA Kit	E.Z.N.A Mag‐Bind Stool DNA Kit	Mag Beads Fast DNA Kit for Feces
Manufacturer details	QIAGEN, Germany	Beaverbio, China	Sangon Biotech, China	TIANGEN Biotech, China	Omega Bio‐Tek, USA	MP Biomedical, USA
Kit name abbreviation	Q Kit	H Kit	S Kit	T Kit	O Kit	M Kit
Separation method	Silica membrane	Magnetized beads	Magnetized beads	Magnetized beads	Magnetized beads	Magnetized beads
Operating steps	14	10	11	13	22	17
RNase A	No	No	Yes	Yes	No	Yes
Protease K	Yes	Yes	No	No	Yes	No
Bead‐beating	No	No	No	Yes	No	Yes

### 2.3 Assessment of DNA Purity and Concentration

The purity of DNA was assessed based on the absorbance readings at 230, 260, and 280 nm, and calculated OD260/280 and OD260/230 ratios, using the NanoDrop One (Thermo Fisher Scientific, USA). Concentration of DNA samples was estimated using the Qubit 4.0 fluorometer (Thermo Fisher Scientific, USA) with Qubit 1 × dsDNA HS Assay Kit (Thermo Fisher Scientific, USA).

### 2.4 STR Analysis

11 STR loci (GPL‐8, GPL‐28, GPL‐29, GPL‐31, gpy‐5, Panda‐05, Panda‐40, GP‐01, GP‐08, GP‐901 and gpy‐20) were selected to evaluate the six DNA extraction kits (summarized in Table 2) (Zhang et al. 1995; Shen et al. 2010; Huang et al. 2015; National Forestry and Grassland Administration 2017). Blood DNA genotypes were used as the gold standard for evaluating the accuracy of fecal DNA genotyping. All PCR reactions were performed in singleplex, with each reaction targeting a single STR locus using a specific pair of primers. Consensus profiling, a method requiring multiple independent PCR replicates per locus to confirm genotypes, was used in this study to determine the genotype of fecal DNA samples at each STR locus (Navidi et al. 1992; Pierre et al. 1996). For each fecal DNA sample, at least three PCRs per locus were analyzed. We also used the Micro‐Checker software to identify and correct genotyping errors caused by null alleles, large allele dropout, and the scoring of stutter peaks (Oosterhout et al. 2004). If a correct genotype from a fecal DNA sample appears at least twice, it is considered to match the genotype of blood DNA of the same individual.

**TABLE 2:** Characterizations of STR loci of giant panda.

STR locus	Primer sequences (5′‐3′)	Accession no.	Repeat motif	Fluorescent dyes	PCR product size range (bp)
GPL‐8	F: TGGTTTTGCAAGGATGACAG R:TTGTGACAAGCAAGCTCCAC	KF907132	(ATCC)11	HEX	224–236
gpy‐5	F: CTCGGGAGCTTTGTACCATC R: CAGAGAGCCCAAACCTCAAC	KF907157	(AACT)16	HEX	200–212
GPL‐28	F: GAAAGAAGGGCAGGGATAGG R: TGACCAAGAACTCACGGTTG	KF907138	(ATAA)21	FAM	186–194
GPL‐29	F: TCCAAGGCTTCAAACAAGGT R: CACCACAGGTGCCAATTATG	KF907139	(ATCC)19	TAMRA	155–179
gpy‐20	F: GCAGGCACTCAAGAGGTGTT R: CCTTGTGCTAAACACAGGTGA	KF907159	(TTTG)16	TAMRA	149–165
GP‐901	F: AGCTAATTTTCCAAGTTACCTTTCC R: GGATCTGGGTGTTATTTGCAATG	—	(CA)23	FAM	156–164
GP‐08	F: AACATCCTGGGTATTCTCCATGC R: TGCAGAGTGAGGACCTAGGTGTC	—	(AC)16GCAC	FAM	154–160
GP‐01	F: ACGGGAAGCCTGCTTCTACACTC R: AGACACCCAACCGACTAAACCAC	—	(CA)13A(CA)2	FAM	144–152
GPL‐31	F: GCATCCTTGTCCTCTTGGAG R:GCATTGTTTTCTACTCTACAAATATCC	KF907141	(ATCT)21	FAM	127–147
Panda‐40	F: CCTACCTATTTACCTACTTACCTACC R: GATGCTATTAAGCAACAGAC	AY161213	(TATC)11	FAM	119–129
Panda‐05	F: GCAAGGATTCATAACGGTAGGG R: GTGTCTTGACCACCAGTGATGTAAGCCG	AY161178	(GT)20	FAM	98–108

All PCR reaction mixtures (20 μL) were prepared as previously described (Kou et al. 2022). The volume of fecal DNA sample template in the PCR reaction system to be tested was 9 μL, and the amount of blood DNA template added in the positive PCR reaction system was 20 ng. PCR was performed on a DNA thermal cycler with the following conditions: initial denaturation at 94°C for 3 min and 40 cycles of 94°C for 10 s, annealing at Tm of each primer for 15 s, 72°C for 30 s, followed by a final extension for 10 min at 72°C. Negative controls (template was replaced by water) of STR loci were used for quality control. The amplification products were separated with capillary electrophoresis using an ABI 3530xl Genetic Analyzer (Thermo Fisher Scientific). All genotyping data was analyzed using GeneMapper ID‐X Software v1.0 (Thermo Fisher Scientific).

### 2.5 Data Statistics

To explore the correct genotyping probability of each STR locus, we calculated the genotype matching rate (GMR) for each extraction kit, *which is the number of* matched fecal DNA samples (same genotype for both fecal DNA and blood DNA) divided by the number of fecal DNA samples (*n* = 5). To evaluate the amplification performance of the 11 STR genotyping systems, we calculated the total genotype matching rate (TGMR), which is the number of matched fecal DNA samples (fecal DNA and blood DNA have the same genotype) in all kits divided by the total number of fecal DNA samples (*n* = 30). *In order to* compare the differences in the ability of the extraction kits to obtain high‐quality host DNA fragments, we also calculated the *amplification success rate (ASR), which is the number of* STR loci *with a* GMR *of 100% in that kit divided by the total number of* STR loci (*n* = 11). The expected intensity of the CE signal (the peak height at the allele position), measured in Relative Fluorescence Units (RFU) is proportional to the number of product amplicons (Karkar et al. 2019). Therefore, we detected the signal intensity of allele peaks by analyzing the peak heights of homozygous peaks or smaller heterozygous peaks to assess the performance differences of each DNA extraction kit. Multiple comparisons were conducted using one‐way analysis of variance (for parametric data) or Kruskal–Wallis (for nonparametric data) tests in R (version 4.3.1). Correction for multiple comparisons was performed using Tukey's HSD test for parametric data and Dunn's test for nonparametric data. A *p* value less than 0.05 was considered statistically significant.

## 3 Results

### 3.1 DNA Extraction Efficiency Varies Across Kits

**FIGURE 2:**
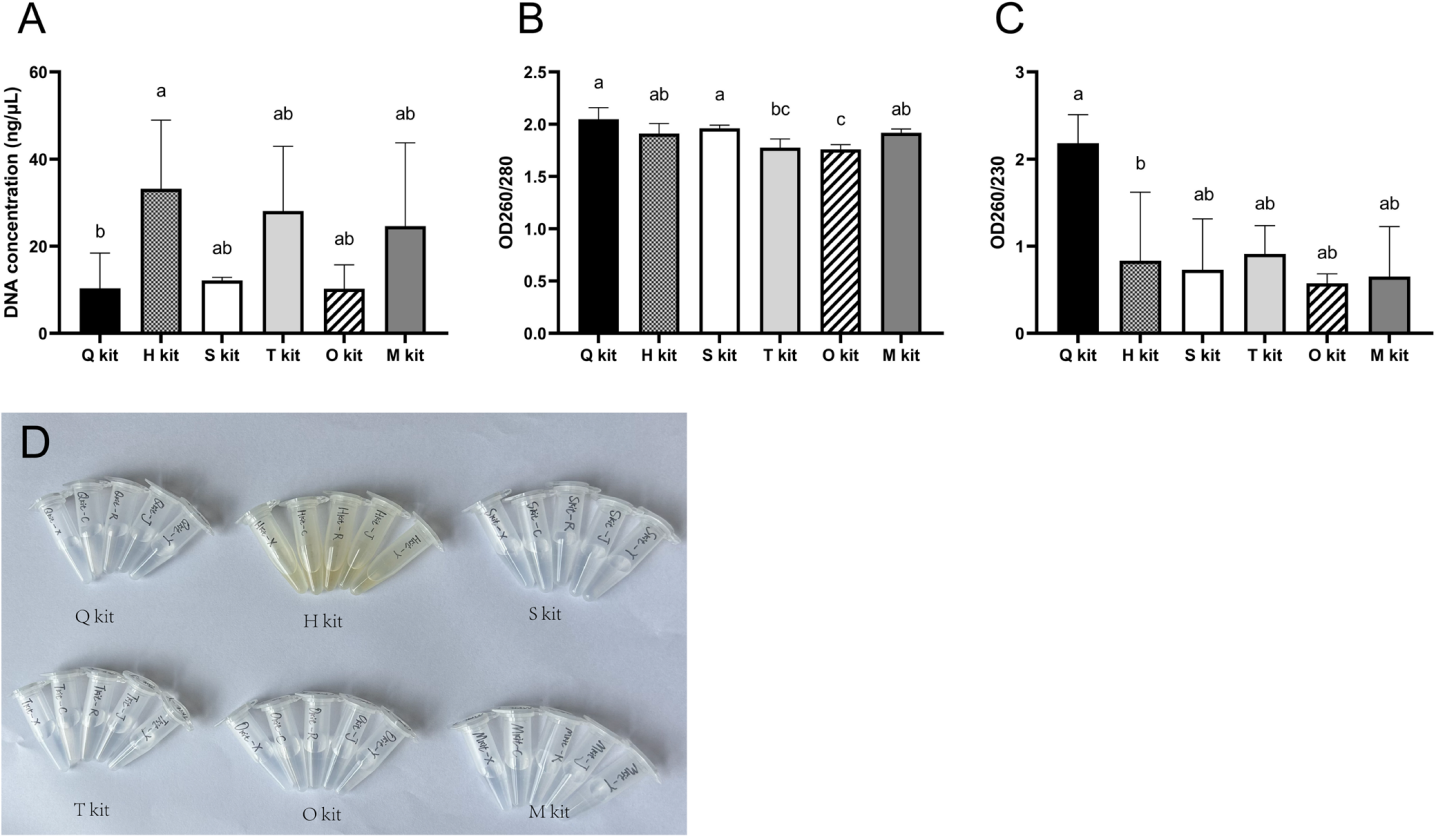
Comparison of the quality of extracted DNA via the QIAamp Fast DNA Stool Mini Kit (Q kit), the Beaver Beads Stool DNA Kit (H kit), the Mag‐MK Soil & Stool Genome DNA Extraction kit (S kit), the Magnetic Soil And Stool DNA Kit (T kit), the E.Z.N.A Mag‐Bind Stool DNA Kit (O kit) and the Mag Beads Fast DNA Kit for Feces (M kit): (A) DNA concentration comparison, (B) DNA purity (OD260/280) comparison, (C) DNA purity (OD260/230) comparison, (D) DNA elution color comparison.

We compared the DNA extraction results of six commercial extraction kits and obtained a total of thirty DNA extraction products. The results of DNA concentration, OD260/280, OD260/230, and DNA elution color are shown in Figure 2. In the present study, H kit produced a significantly higher amount of DNA from fecal samples compared with Q kit (mean ± standard deviation: Q kit = 10.31 ± 8.13 ng/μL, H kit = 33.19 ± 15.77 ng/μL, S kit = 12.13 ± 0.74 ng/μL, T kit = 28.09 ± 14.87 ng/μL, O kit = 10.25 ± 5.47 ng/μL, M kit = 24.62 ± 19.14 ng/μL, *p* < 0.05). DNA purity, measured by both OD260/280 and OD260/230, was found to be optimal in Q kit. All fecal DNA samples fulfilled the purity criterion of OD260/280 (mean values within a range of 1.75–2.04 ng/μL), whereas as a secondary measure of DNA purity, the OD260/230 values of DNA samples from H kit (mean value = 0.83 ng/μL) were significantly lower than those from Q kit (*p* < 0.05, mean value = 2.18 ng/μL). The DNA elution colors varied among different kits. The DNA elution from the H kit was light yellow or light brown, while the DNA elution from other kits was transparent.

### 3.2 DNA Extraction Kit Affects the Amplification Performance of STR Genotyping Systems

**FIGURE 3:**
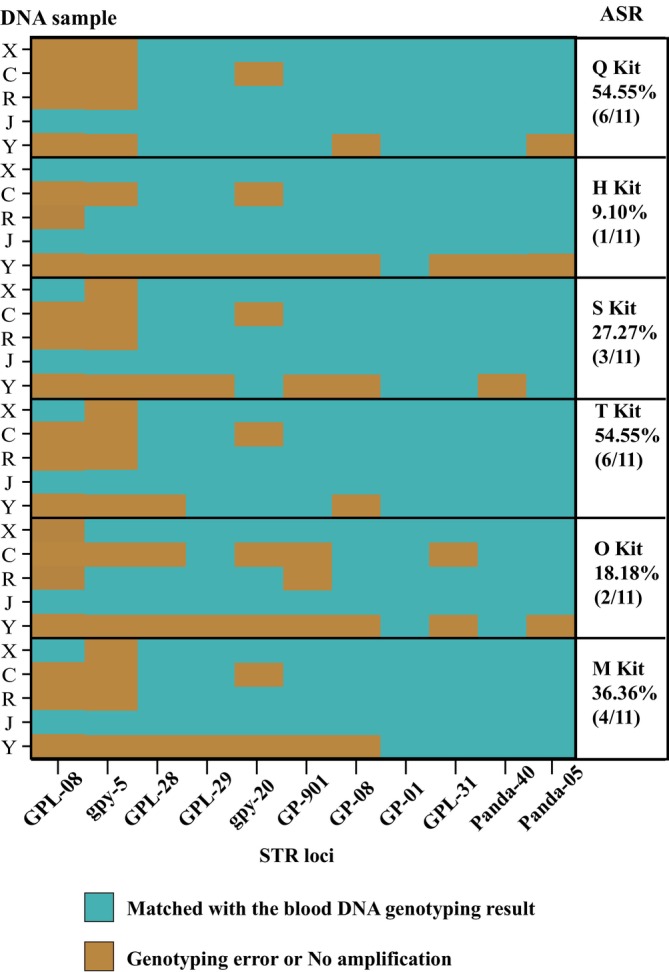
Genotyping correctness at each STR locus. “ASR” means amplification success rate.

Thirty DNA samples from six commercial extraction kits were genotyped by using 11 STR genotyping systems, and the results are shown in Table 3 and Figure 3. The TGMR varied across different STR loci, which suggests that there are differences in the amplification performance of different STR genotyping systems without considering the DNA extraction kits. For STR genotyping systems (GPL‐08 and gpy‐5) with longer PCR product sizes (greater than 200 bp), the TGMR was only 33.33%. On the contrary, for STR genotyping systems (gpy‐20, GPL‐28, GPL‐901, GP‐08, GPL‐29, Panda‐05, GPL‐31 and Panda‐40) with smaller PCR product sizes (less than 200 bp), the TGMR reached 70.00%–93.33%, whereas the TGMR resulted in 100% when used with the STR genotyping system GP‐01.

**TABLE 3:** The amplification performance of STR genotyping systems.

Parameter	Kit name abbreviation	GPL‐08	gpy‐5	GPL‐28	GPL‐29	gpy‐20	GP‐901	GP‐08	GP‐01	GPL‐31	Panda‐40	Panda‐05
GMR	Q kit	20% (1/5)	20% (1/5)	100% (5/5)	100% (5/5)	80% (4/5)	100% (5/5)	80% (4/5)	100% (5/5)	100% (5/5)	100% (5/5)	80% (4/5)
	H kit	40% (2/5)	60% (3/5)	80% (4/5)	80% (4/5)	60% (3/5)	80% (4/5)	80% (4/5)	100% (5/5)	80% (4/5)	80% (4/5)	80% (4/5)
	S kit	40% (2/5)	20% (1/5)	80% (4/5)	80% (4/5)	80% (4/5)	80% (4/5)	80% (4/5)	100% (5/5)	100% (5/5)	80% (4/5)	100% (5/5)
	T kit	40% (2/5)	20% (1/5)	80% (4/5)	100% (5/5)	80% (4/5)	100% (5/5)	80% (4/5)	100% (5/5)	100% (5/5)	100% (5/5)	100% (5/5)
	O kit	20% (1/5)	60% (3/5)	60% (3/5)	80% (4/5)	60% (3/5)	40% (2/5)	80% (4/5)	100% (5/5)	60% (3/5)	100% (5/5)	80% (4/5)
	M kit	40% (2/5)	20% (1/5)	80% (4/5)	80% (4/5)	60% (3/5)	80% (4/5)	80% (4/5)	100% (5/5)	100% (5/5)	100% (5/5)	100% (5/5)
TGMR	—	33.33% (10/30)	33.33% (10/30)	80% (24/30)	86.67% (26/30)	70.00% (21/30)	80% (24/30)	80% (24/30)	100% (30/30)	90.00% (27/30)	93.33% (28/30)	90% (27/30)

Abbreviations: GMR, genotype‐matching rate; TGMR, total genotype matching rate.

However, for some STR genotyping systems, the choice of DNA extraction kit can affect the genotyping performance, which suggests that the ability of different DNA extraction kits to obtain high‐quality target DNA varies. For GPL‐08, H, S, T, and M kits had a GMR of 40%, but 20% for Q and O kits. For gpy‐5, H and O kits had a 60% GMR, while other extraction methods reached 20%. For GPL‐28, GP‐901, and GPL‐31, the GMRs of the O kit were lowest among all kits (60%, 40% and 60%, respectively). Regarding GPL‐29, only Q and T kits reached a 100% GMR, with the other kits at 80%. For gpy‐20, the GMRs of H, O, and M kits reached 60%, while only the Q kit reached a 100% GMR, with S and T kits at 80%. In contrast, for all kits, GP‐08 achieved a GMR of 80%, whereas GP‐01 reached a 100% GMR. Similarly, for Panda‐40 and Panda‐05, GMRs were more than 80% in all kits.

We further evaluated the ASR values across various kits to determine the most suitable DNA extraction kit for STR genotyping systems used in the present study. The results (Figure 3) showed that the Q and T kits exhibited the highest ASR at 54.55%. In contrast, the M, S, and O kits had relatively low ASRs of 36.36%, 27.27%, and 18.18%, respectively, while the H kit demonstrated the lowest ASR at 9.10%.

### 3.3 Performance Differences of DNA Extraction Kits

**FIGURE 4:**
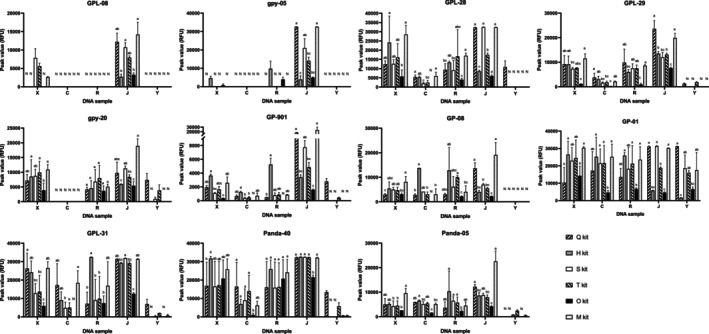
The comparison of allelic peak intensities. Error bars represent ± one standard deviation (SD) from the mean of DNA extraction products from five individuals. Lowercase letters (a–c) indicate statistically significant differences (*p < 0.05*) based on Tukey's HSD test for parametric data or Dunn's test for nonparametric data, and groups sharing the same letter are not significantly different. Capital letter “N” denotes a genotyping mismatch including genotyping error or no amplification.

We analyzed the allele peak intensity of matched fecal DNA samples to further understand the performance differences of each DNA extraction kit. The results (Figure 4) showed that the allele peak intensities of the matched fecal DNA samples extracted with the O kit were more likely to be low compared to those of the other extraction kits, which indicated that the efficiency of obtaining the high‐quality target DNA fragments with the O kit is relatively low.

## 4 Discussion

The combination of noninvasive fecal DNA and STR genotyping minimizes disturbance to endangered wild giant pandas while providing valuable data on individual identification and population genetic diversity. However, low‐quality fecal DNA may result in PCR amplification failure or genotyping errors, thereby compromising the reliability of individual identification based on STR profiles. To address this challenge, we evaluated the impact of six commercial fecal DNA extraction kits on the amplification performance of 11 STR genotyping systems. Using a consensus profiling approach (Navidi et al. 1992; Pierre et al. 1996; Taberlet et al. 1998), in combination with a pre‐optimized PCR reaction system and amplification protocols (Kou et al. 2022), we systematically compared genotypes obtained from paired blood and fecal samples of giant pandas. This comparative assessment aims to enhance the reliability of STR genotyping results based on fecal DNA and provide practical insights for standardizing methodologies in noninvasive genetic research.

In the present study, the six DNA extraction kits varied in fecal DNA concentration and purity. All DNA samples had mean OD260/280 values in the range of 1.75–2.04 ng/μL, suggesting “pure” DNA. However, only the Q kit extracted DNA with an acceptable mean OD260/230 value, indicating superior removal of PCR inhibitors. Low OD260/230 values indicate the presence of PCR inhibitors absorbing at 230 nm, such as polysaccharides, EDTA, salts, or other aromatic compounds (Lucena‐Aguilar et al. 2016). Moreover, the DNA samples from the H kit ranged in color from light yellow to light brown, indicating that the H kit had a poor ability to remove pigments of bamboo from fecal residues. Therefore, extracting DNA by H, S, T, O, and M kits might contain PCR inhibitors that could interfere with downstream applications. The Q kit produced purer fecal DNA but lower total DNA concentration, which is consistent with previous studies (Hart et al. 2015; Claudel et al. 2021; Srirungruang et al. 2022). Although the H kit provided higher total DNA concentrations than the Q kit (*p* < 0.01), it performed worse in ASR values. Our results highlight that high fecal DNA concentration alone does not ensure successful PCR amplification. Other factors, including DNA purity (OD260/280 and OD260/230 values) and DNA elution color, must also be considered to achieve reliable STR genotyping results.

In microbiome research, a major source of experimental variation is the lack of standardized methods for fecal DNA extraction (Fernández‐Pato et al. 2024). The abundance of host cells is much lower than that of microbial cells in fecal samples, and most commercial fecal DNA extraction kits are optimized for long microbial genomic DNA rather than short host DNA fragments (He et al. 2019). Some studies suggest that screening DNA extraction kits is crucial for host DNA research as well (Vandenberg and van Oorschot 2002; Johnson et al. 2005; Teixeira et al. 2015; He et al. 2019). Usually, when the animals' diet is rich in easily digestible fibers, the digestion process may be less abrasive to the intestinal epithelium, ultimately affecting the number of epithelial cells per gram of fecal sample (Costa et al. 2016). The diet of Giant pandas, rich in bamboo fibers, affects the composition of their feces, making fecal host DNA extraction more challenging. This highlights the need to carefully consider DNA extraction kits for giant pandas. Until now, there have been no studies comparing differences in the ability of any commercial DNA extraction kits to extract giant panda host DNA. In the present study, we found that the Q and T kits were more effective for the extraction of host DNA from giant panda fecal samples for 11 STR genotyping systems based on the ASR results. However, the performance of the Q kit and T kit may not be universally applicable to all STR genotyping systems developed for the giant panda. We also found that there were differences in the ability of the DNA extraction kits to obtain high‐quality host DNA fragments, resulting in different GMR values. For some STR genotyping systems with longer PCR product sizes (greater than 200 bp), fecal DNA extraction kits should be carefully selected. For example, the H kit and O kit may be more suitable for gpy‐5, while the use of the Q kit and O kit should be avoided when using GPL‐08. For STR genotyping systems with smaller PCR product sizes (GPL‐29, GP‐08, GP‐01, Panda‐40 and Panda‐05), choosing any of the six kits guaranteed a high probability of correct genotyping (GMR values were more than 80%). In the present study, we did not test for the presence of PCR inhibitors in fecal DNA samples, but rather compared the suitability of the kits by reflecting the overall quality of the template DNA through the match rate between observed and true genotypes of individuals. Small amounts and/or degradation of DNA can lead to allele peaks that may be difficult to distinguish from background noise peaks, affecting the determination of individual genotypes (Karkar et al. 2019). Therefore, factors that contribute to a decrease in the overall quality of the target DNA (e.g., use of long exposed fecal samples) may reduce the allele peak intensities, resulting in a decrease in the suitability of the DNA extraction kits for use in some STR genotyping systems. It is worth noting that the complex composition of our fecal samples, which contain bamboo leaf and culm residues, and only a small number of STR genotyping systems were used to evaluate the efficiency of the kit in recovering host DNA. As test kits may be optimized for different types of fecal samples or for the diversity of DNA fragments recovered, kits that performed poorly in this study may show excellent performance when used for less complex samples (e.g., fecal samples containing only bamboo shoot residue) or for other STR genotyping systems that we did not use.

Consistent with previous studies (Pompanon et al. 2005; Zhang et al. 2018; Forgacs et al. 2019), we found that different STR loci exhibited varying genotype matching rates within the same batch of DNA samples. We also found that the STR genotyping systems with longer PCR product sizes (GPL‐08 and gpy‐5 in this study) were less likely to yield high TGMR values. Differences in amplification efficiency of the STR genotyping systems and degradation of the target DNA may explain these results. Previous studies indicated that even with high‐quality DNA from noninvasive samples, some STR loci are more prone to genotyping errors, particularly those with larger PCR products (Bonin et al. 2004; Hoffman and Amos 2005). Moreover, according to the model of random degradation of DNA, the amount of amplifiable fecal host DNA was inversely related to PCR product size (Deagle et al. 2006). In population genetics studies and individual identification studies of giant pandas, 6–15 STR genotyping systems are commonly used. Low genotype error rates are crucial for ensuring the accuracy and efficiency of genetic analysis. Therefore, selecting STR genotyping systems with appropriate amplicon sizes and then matching them with the appropriate DNA extraction kit is key to minimizing multi‐locus genotyping mismatches. Due to the lack of additional blood DNA samples for pairwise validation and the limited number of STR loci tested, further studies of the link between giant panda STR loci selection and the fecal DNA extraction kit selection are needed.

In conclusion, the Q and T kits were the most efficient fecal host DNA extraction kits for STR genotyping systems in the present study, and the Q kit had a greater ability to remove PCR inhibitors than other kits. The present study also emphasizes the need to select the most suitable kit based on the STR genotyping systems used to achieve the highest possible genotyping correctness. For STR genotyping systems with smaller PCR product sizes (less than 200 bp, GPL‐29, GP‐08, GP‐01, Panda‐40 and Panda‐05 in the present study), choosing any of the six kits guaranteed a high probability of correct genotyping. For GPL28, GP901, and GPL31, the O kit should be avoided, while for gpy20, both H and O kits should be avoided to ensure GMR > 80%. However, the fecal DNA extraction kit should be carefully selected with caution for STR genotyping systems with longer PCR product sizes (greater than 200 bp, GPL‐8 and gpy‐5 in the present study).

## Author Contributions

**Jie Gao:** conceptualization (lead), data curation (equal), formal analysis (equal), methodology (equal), project administration (lead), visualization (equal), writing – original draft (equal), writing – review and editing (equal). **Chunhai Li:** conceptualization (equal), data curation (lead), formal analysis (lead), methodology (lead), project administration (equal), visualization (equal), writing – original draft (equal), writing – review and editing (equal). **Yitao Wu:** data curation (equal), formal analysis (equal), visualization (equal), writing – review and editing (supporting). **Xinyong Zhu:** data curation (equal), formal analysis (equal), writing – review and editing (equal). **Siqin Liu:** data curation (equal), formal analysis (supporting), writing – original draft (supporting). **Yang Zhang:** data curation (equal), writing – original draft (supporting). **Huizhong Pang:** visualization (supporting), writing – original draft (equal), writing – review and editing (equal). **Jiaheng Li:** visualization (equal), writing – review and editing (equal). **Jiawen Liu:** data curation (supporting), resources (supporting), writing – original draft (equal), writing – review and editing (supporting). **Wangsheng Zhao:** resources (supporting), supervision (equal), writing – original draft (supporting), writing – review and editing (equal). **Ye Wang:** data curation (supporting), resources (equal), supervision (equal), writing – original draft (supporting), writing – review and editing (equal). **Jie Kou:** data curation (supporting), formal analysis (equal), funding acquisition (lead), methodology (equal), resources (equal), writing – original draft (supporting), writing – review and editing (equal).

## Ethics Statement

This work was conducted according to the guidelines of the Chengdu Research Base of Giant Panda Breeding Institutional Animal Care and Use Committee (protocol code 2020015 and 2021006).

## Conflicts of Interest

The authors declare no conflicts of interest.

## Data Availability

The authors declare that the data supporting the findings of this study are available within the paper. Data generated from this study are detailed in the text or associated figures and tables.
